# Indigenous *Lactococcus lactis* with Probiotic Properties: Evaluation of Wet, Thermally- and Freeze-Dried Raisins as Supports for Cell Immobilization, Viability and Aromatic Profile in Fresh Curd Cheese

**DOI:** 10.3390/foods11091311

**Published:** 2022-04-30

**Authors:** Justina Mileriene, Loreta Serniene, Kristina Kondrotiene, Valentini Santarmaki, Yiannis Kourkoutas, Agne Vasiliauskaite, Lina Lauciene, Mindaugas Malakauskas

**Affiliations:** 1 Department of Food Safety and Quality, Veterinary Academy, Lithuanian University of Health Sciences, Tilzes St. 18, LT-47181 Kaunas, Lithuania; loreta.serniene@lsmuni.lt (L.S.); kristina.kondrotiene@lsmuni.lt (K.K.); agne.vasiliauskaite@lsmu.lt (A.V.); lina.lauciene@lsmuni.lt (L.L.); mindaugas.malakauskas@lsmuni.lt (M.M.); 2 Laboratory of Applied Microbiology & Biotechnology, Department of Molecular Biology & Genetics, Democritus University of Thrace, 68100 Alexandroupolis, Greece; vsantar@mbg.duth.gr (V.S.); ikourkou@mbg.duth.gr (Y.K.)

**Keywords:** indigenous *Lactococcus lactis*, freeze-drying, thermal drying, immobilization, raisins, curd cheese, survival, aroma profile

## Abstract

Indigenous *Lactococcus lactis* enriched raisins were incorporated in fresh curd cheese in wet, thermally dried, and freeze-dried form to produce a novel probiotic dairy product. Symbiotic cheese represents a rising trend in the global market. The viability of *L. lactis* cells was assessed in the cheeses during storage at 4 °C for 14 days and the effect of the added enriched raisins on physicochemical parameters, microbiological characteristics, and sugar content, aromatic profile, and sensory acceptance of cheeses were evaluated. Immobilized *L. lactis* cells maintained viability at necessary levels (>6 log cfu/g) during storage and significantly increased the acceptability of cheese. The addition of raisins enhanced the volatile profile of cheeses with 2-furanmethanol, 1-octanol, 3-methylbutanal, 2-methylbutanal, 2-furancarboxaldehyde, 1-(2-furanyl)-ethanone, 5-methyl-2-furancarboxaldehyde. The obtained results are encouraging for the production of novel fresh cheeses with improved sensorial and nutritional characteristics on industrial and/or small industrial scale.

## 1. Introduction

Concerned by the rising number of life-threatening chronic illnesses in western countries, consumers are not only more careful about their food selections but also focusing their attention on healthy food [1]. Responding to the consumer needs, dairy producers are constantly searching for innovations, which have led to the wide use of probiotics in various dairy products, targeting fermented milk at first, and invading various cheese productions now. The latest trend in this area is the usage of wild-type, indigenous novel presumptive probiotics isolated from various local sources [2,3]. They are being extensively investigated as possible starters, protective cultures, nutraceuticals, probiotics, and flavor enhancers [4,5,6]. Flavor compounds, such as lactones, carboxylate esters, alcohols, ketones, aldehydes, and pyrazines produced by microbes, are some of the most important attributes to cheese. Lactate and lactose metabolism are the key routes for volatile flavor compound (VFC) synthesis in cheese, which are highly dependent on the cheese variety, bacteria used, and storage conditions [7].

In our previous study, we aimed to characterize indigenous *Lactococcus lactis* strains, previously isolated from food-grade samples, through the measure of their protective, technological, and probiotic properties among others [8]. However, supplementation of curd cheese with LAB cultures can be problematic, especially when high curd temperature is an essential part of the manufacturing process to form the uniform cheese body.

Fresh acid whey, a byproduct of cottage cheese production, is gradually added to boiling milk to coagulate protein in traditional East European sweet curd cheese production. High temperature applied in the process prevents the significant loss of valuable whey proteins; however, a high temperature of ready-to-be pressed curd might threaten the survival of mesophilic bacteria cells. Therefore, immobilization and the addition of prebiotics as a support are widely used to improve probiotic cell viability while acting as a protective shield against thermal and acid stress [9,10]. Prebiotics can increase the survival rate and stability of probiotics during processing and storage, especially when they are used as the carrier for the immobilization of probiotic strains [11,12]. 

Raisins (dried *Vitis vinifera* L. grapes) are known to be rich in both single sugars (fructose and glucose) and non-digestible fructo-oligosaccharides (FOS) (1–5%) [13]. Raisins contain significant amounts of tartaric, proto-catechuic, and oxidized cinnamic acids, flavanols, such as quercetin and kaempferol, and phenolic acids, such as caftaric and coutaric acid compared to their hydrated counterparts [14], as well as fermentable fibers, such as inulin-type fructans that affect gut microbiota composition, thus being a good source of prebiotic functional food [15,16,17,18].

The aim of the study was to evaluate the impact of *Lactococcus lactis* strain immobilized on raisins on quality parameters of traditional sweet curd cheese during refrigerated storage. Therefore, the objectives of this study were as follows: (1) to produce wet, thermally, and freeze-dried immobilized cultures of indigenous *Lactococcus lactis* strain with presumptive properties on raisins; (2) to supplement traditional sweet curd cheese with *Lactococcus lactis* strain immobilized on raisins; and (3) to evaluate the effects of this supplementation on the physicochemical, microbiological, and sensory parameters of the obtained cheese during its refrigerated storage. 

## 2. Materials and Methods

### 2.1. Microorganisms and Materials

*Lactococcus lactis* subsp. *lactis* LL16, previously isolated from raw bovine milk [19] before the use in this study was stored at −80 °C in M17 broth (Merck, Darmstadt, Germany) in the presence of 30% glycerol. *L. lactis* LL16 strain for this experiment was selected as a promising probiotic, protectant, and flavor-enhancing strain [6]. Before immobilization, *L. lactis* LL16 was revitalized in M17 broth (Biolife, Milano, Italy) by growing for 48 h at 30 °C. The biomass of *L. lactis* was harvested by centrifugation at 4000 rpm for 15 min at 4 °C. 

Sun-dried Corinthian raisins (*Vitis vinifera* L.) [20] with no additives (Couniniotis Group, Meganitis Bridge, Egio, Greece) were purchased from a local supermarket. The chemical composition of raisins used in this study was as follows: fat (0.4%), carbohydrates (67.8%), fiber (5.9%), and protein (2.3%). In order to reduce the initial microbiological load of raw materials, the raisins were sterilized at 121 °C for 15 min prior to use. 

Pasteurized bovine milk (pH 6.6, 4.0% fat, 3.0% protein, 4.5% lactose, 8.1% non-fat solids, determined with Milko-Skan, FOSS ELEKTRIK, Hillerød, Denmark) was purchased from the local supermarket. Fresh acid whey (5.0 pH, 0.3% fat, 0.9% protein, 3.8% lactose, 6.4% dry matter, determined with Milko-Skan, FOSS ELEKTRIK, Hillerød, Denmark) was produced by heating fermented milk (purchased from a local supermarket) at 45 °C for 15 min and straining obtained acid whey after protein aggregation occurred due to heat and acidity. 

### 2.2. Methods

#### 2.2.1. Immobilization of *L. lactis* LL16 on Raisins

Immobilization of *L. lactis* on raisins was based on the study by Bosnea et al. [21] with modifications. In brief, after sterilization, ¼ of raisins (50 g) were set aside (first treatment), while the other three parts (3 × 50 g) were transferred to beakers with ¼ Ringer’s solution (VWR Chemicals, Poole, UK) and *L. lactis* LL16 biomass at a ratio of 45 mL/1.5 g (wet weight) of cells (which accounted for 9 log cfu/mL bacterial concentration) and kept for 2 h at room temperature. After that, the solution was decanted. Wet immobilized cultures on raisins from one of the beakers were put into a sterile air-tight container and stored at 4 °C for 24 h (second treatment). The other parts were thermally dried for 24 h in 30 °C (third treatment) or were frozen overnight at −80 °C and then freeze-dried in a BenchTop Pro (Virtis, SP Scientific, Warminster, PA, USA) for 12 h at ~30–35 Pa with the condenser temperature fixed at −101 °C (fourth treatment). Graphical illustration of *L. lactis* immobilization is presented in Supplementary file Figure S2.

#### 2.2.2. Manufacture of Experimental Curd Cheese

Sweet curd cheese was made at the laboratory by a conventional method with some modification as follows: bovine milk was heated to 90 °C and then fresh acid whey was gradually added until full coagulation of curd was attained. Then the curd was put into a metal strainer and left to drain and cool for 30 min. The resultant curd (19.0% fat, 24.6% protein, 2.2% lactose, 48.6% dry matter, determined with FoodSkan (FOSS, Hillerød, Denmark), pH 5.6 (pH-meter HI 211)) was used as a base to produce the control and experimental curd cheese samples: plain curd cheese (control, C), curd cheese with 1% *L. lactis* LL16 biomass (free cells, C + FC), curd cheese with 20% of raisins (C + R), curd cheese samples with immobilized *L. lactis* LL16 cells on raisins (20% of wet, C + RW; 17.4% of thermally-dried, C + RTD; 17.6% of freeze-dried, C + RFD). The resultant cheese samples (150 g) in triplicates were packed in air-tight containers and stored in a refrigerator at 4 ± 1 °C for 14 days for further analysis. Graphical illustration of manufacture of experimental curd cheese is presented in Supplementary file Figure S1.

#### 2.2.3. Physicochemical Analysis

Samples for all the subsequent analyses were taken in triplicates on storage days 1, 7, and 14. Water activity (a_w_) was determined using the HygroLab 3 (Rotronic AG, Bassersdorf, Switzerland), according to the manufacturer’s guides. The pH was measured using a pH-330i pH meter (WTW GmbH, Gotha, Germany). Moisture content was calculated after the determination of total solids according to the oven method described in ISO: 5534 [22].

#### 2.2.4. Microbiological Analysis

Samples of raisins (before and after immobilization process) were tested to determine the counts of lactococci: 10 g were diluted (1:10, *w*/*v*) with peptone water solution (Liofilchem, Roseto, Italy), mixed, submitted to 10 decimal serial dilutions, and plated on M17 agar (Oxoid, Hampshire, UK) supplemented with 10% lactose solution (Duchefa Biochemie, Haarlem, The Netherlands) after 48 h incubation at 37 °C.

For microbiological analysis of cheeses, samples in triplicates were taken at storage days 1, 7 and 14, diluted (1:10, *w*/*v*) with peptone water solution (Liofilchem, Roseto, Italy), mixed, and submitted to 10 decimal serial dilutions. Following standard ISO methods [23], the quantifications of microbiological counts were determined on the selective media for each species. Viable counts of total aerobic bacteria (TBC) were enumerated on plate count agar (PCA) (Oxoid, Hampshire, UK) after 48 h incubation at 30 °C. Lactococci counts were enumerated on M17 agar (Oxoid, Hampshire, UK) supplemented with 10% lactose solution (Duchefa Biochemie, The Netherlands) after 48 h incubation at 37 °C. *Staphylococcus* spp. were enumerated on Baird-Parker Agar Base supplemented with egg yolk (Biolab, Budapest, Hungary) after 48 h incubation at 37 °C. Enterobacteria cultured in Violet Red Bile Glucose Agar (Lab M Ltd., Heywood, UK) was enumerated after 24 h at 37 °C. Coliforms were enumerated on Violet Red Bile Agar (Lab M Ltd., Heywood, UK) after 24 h at 30 °C. Yeast and molds were enumerated on acidified with sterile lactic acid MALT Extract Agar (pH 4.5) (Lab M Ltd., Heywood, UK) to inhibit bacteria growth after incubation at 30 °C for 72 h followed by microscopic confirmation test for each type of colony encountered. 

#### 2.2.5. Sugar Profile, Organic Acids, Glycerol, and Ethanol

High-performance liquid chromatography (HPLC) was used to determine sugars profile (lactose, galactose, glucose, fructose, and total sugars), organic acids (citric, lactic, acetic, tartaric, butyric), glycerol, and ethanol concentrations, as previously described by Prasanna et al. [24]. Briefly, each sample (5 g) was homogenized with 5 mL of ultrapure water, 1.2 mL of 85% trichloroacetic acid (TCA) was added and the mixture was then stored at 4 °C for 24 h for protein precipitation. Subsequently, the samples were centrifuged at 10,000× *g* for 30 min at 4 °C and the supernatant was filtered using 0.2 μm-filters. The HPLC system consisted of a Shimadzu chromatograph with a NUCLEOGEL 300 OA, 300 × 7.8 mm ion exchange column (Macherey-Nagel, Allentown, PA, USA), coupled with a guard column, an LC-20AD pump (Shimadzu, Dusseldorf, Germany) and a CTO-20AC oven (Shimadzu) operated at 70 °C. The target compounds were detected using a RID-10A refractive index detector. Solution of 0.001 N sulphuric acid was used for elution at a flow rate of 0.3 mL/min. Sugars and organic acids concentrations were determined based on standard curves.

#### 2.2.6. Volatile by-Products

Minor volatile by-products were determined at 1 and 14 days of cheese storage by headspace solid-phase microextraction (HS-SPME) gas chromatography-mass spectrometry (GC-MS) analysis, as recently described by Sidira et al. [25] using a GC/MS (6890 N GC, 5973 NetworkedMS MSD, Agilent Technologies, Santa Clara, CA, USA) equipped with an HP-5MS column (30 m, 0.25 mm i.d., 0.25 μm film thickness). In brief, the samples (5 g) were dissolved in 6 mL of saturated NaCl aqueous solution and then placed into a 20-mL headspace vial fitted with a Teflon-lined septum sealed with an aluminum crimp seal, through which an SPME syringe needle (bearing a 2-cm fiber coated with 50/30-mm divinylbenzene-carboxen on polydimethylsiloxane bonded to a flexible fused silica core; Supelco, Bellefonte, PA, USA) was introduced. The container was then kept at 60 °C with a thermostat for 45 min. Helium was used as the carrier gas (linear velocity of 1.5 mL/min). The oven temperature was set at 35 °C for 6 min followed by a temperature gradient increase of 2° C/min to 60 °C and then 10 °C/min to 240 °C. A final extension was applied at 240 °C for 10 min. The injector and detector temperatures were 280 °C and 250 °C, respectively. The injector was operated in splitless mode and the mass spectrometer in the electron impact mode with the electron energy set at 70 eV. Identification was performed by comparison of the retention times and mass spectra with those of standard compounds (in-house libraries) and data obtained from NBS75K and Wiley275 libraries, as well as by determining kovats’ retention indexes by injection of a standard mixture containing the homologous series of normal alkanes (C8–C24) in pure hexane under exactly the same experimental conditions, as described above, and comparing them with those reported in the literature. Semi-quantification of volatile compounds was based on 4-methyl-2-pentanol (Sigma-Aldrich, Schnelldorf, Germany), used as an internal standard (IS). Thus, the volatile compounds were quantified by dividing the peak areas of the compounds of interest by the peak area of the IS and multiplying this ratio by the initial concentration of the IS (expressed in micrograms per kilogram).

#### 2.2.7. Preliminary Sensory Evaluation

Preliminary sensory evaluation for overall acceptability of cheese was performed at 1st and 14th days of storage in a trained panel of 14 individuals, including staff members and assistants. All panelists gave their informed consent for inclusion before they participated in the study. Cheese samples (5 × 2 × 2 cm^3^ blocks) were randomly presented for the panel members in identical plastic plates and identified by a random 3-digit number. Panelists used water to clean their palates between samples. The scorecard for overall acceptability was from 0 (unacceptable) to 10 (extremely acceptable) [26].

### 2.3. Statistical Analysis

Two-way ANOVA with storage time, treatments (free/immobilized cells; raisins /immobilized raisins; and immobilized wet/immobilized dried raisins), and their interaction as factors was employed to carry out data statistical analysis included in SPSS software (SPSS Inc., SPSS 24, Chicago, IL, USA). All analysis were performed using Tukey’s HSD post hock tests with a 95% confidence level, corresponding to a critical *p* = 0.05. The concentrations of volatile compounds (GC–MS) were used as variables in hierarchical cluster (HCA) and principal component (PCA) analysis. Hierarchical cluster analysis was performed using Euclidean distance and Ward’s method in SPSS software (SPSS Inc., USA). XLSTAT 2015.1 was used to compute the principal component analysis algorithm and obtain a PCA score plot.

## 3. Results and Discussion

### 3.1. Effect of Immobilization on Raisins

The effect of immobilization was assessed by determining lactococci counts (as described in Section 2.2.4) on raisins right after the immobilization process (before further drying treatments). Before immobilization, lactococci counts in sterilized raisins were below detectable level (<1 log cfu/g). The immobilization process was successful since wet immobilized cells on raisins ranged in levels >6 log cfu/g. 

### 3.2. Physicochemical and Microbiological Changes

The results of physicochemical (pH, moisture, and a_w_) and microbiological (TBC and lactococci) changes after 1, 7 and 14 days of cheese storage are shown in Table 1. As expected, cheese supplementation with 20% of dry raisins in samples C + R, C + RTD, and C + RFD resulted in significantly lower (*p* < 0.05) moisture content, compared to other samples. *L. lactis* cell-free sample with raisins (C + R) also expressed lowest pH values during storage (*p* < 0.05). This is in agreement with previous observations by Pastorino et al., 2003 [27] in which pH positively correlates to moisture content in cheese. The reduction of pH in raisin supplemented samples was most likely related to detected higher contents (*p* < 0.05) of tartaric acid (Table 2). The nature of *L. lactis* LL16 (free or immobilized cells), the support (raisins/immobilized cells on raisins) and the nature of immobilized cultures on raisins (wet or dried) had a significant (*p* < 0.05) effect on cheese pH, moisture, and lactococci counts. Moreover, the changes in pH and lactococci parameters were significantly affected by the storage time and the interaction of the nature of *L. lactis* LL16 (free or immobilized cells) with storage time.

Among all samples, the addition of 1% (*w*/*w*) of free cells to cheese (C + FC) resulted in the highest lactococci counts (8 to 10 log cfu/g) throughout storage. The addition of 17–20% of immobilized cells on raisins resulted in curd cheeses with significantly higher (1 to 3 log cfu/g) lactococci counts than control cheese. On day 1, among samples with raisins, sample C + RW had the highest lactococci counts. The results indicate that wet cells immobilized on raisins had the highest survival rate compared to use drying methods. This is due to the wet nature of cells immobilized raisins, which were not dried after the immobilization process, which led to a higher cell survival rate. 

Remarkably, a gradual growth in lactococci counts was observed in all samples with immobilized cells throughout the storage, however thermally dried cells expressed the lowest counts, compared to wet and freeze-dried cells (*p* < 0.05). This cell surface damage that occurs during drying may provide an explanation of the results above [28]. Nevertheless, previous studies demonstrate that the addition of raisins or fructo-oligosaccharides as a prebiotic either support the viability of probiotics [29] or enhance their growth [30] in cheese, thus suggesting that the nature of the effect is highly strain and matrix dependent [31,32].

The high values of TBC counts are probably attributed to non-starter lactic acid bacteria (NSLAB), since the microbiological counts of coliforms, enterobacteria, *S. aureus*, and yeasts and molds were below the detectable level (≤1 log cfu/g) in all cheese samples (data not shown). 

### 3.3. Sugars and Organic Acids

The effect of immobilization of *L. lactis* LL16 on raisins compared to free cells on sugar profile and its conversion is presented in Table 2. 

Only the nature of *L. lactis* LL16 (free or immobilized cells) had a significant (*p* < 0.03) effect on sugar profile and organic acids (citric, lactic, acetic, and tartaric acids). Only lactose and traces of galactose were detected in the samples without raisins. Higher total sugar content, as well as the presence of glucose and fructose, was found in all the samples with raisins. Samples C + RW had less glucose and fructose at the 1st storage day compared to C + RTD and C + RFD samples. This is most likely due to the advanced hydrolytic activity of *L. lactis* LL16 during the treatment of raisins: wet immobilized cells on raisins were stored at 4 °C for equal time period (24 h) that it took to prepare thermally and freeze-dried immobilized cells on raisins. The results of our previous study revealed that *L. lactis* LL16 has the ability to hydrolyze various carbohydrates [8].

The fastest conversion of glucose and fructose was reported in C + RTD samples on the 7th day compared to C + RW and C + RFD samples. Lower concentrations of these sugars were observed in C + RTD samples at the end of cheese storage compared to C + RFD sample. The nature of sugar conversion differed between samples with free and immobilized cells. A gradual decrease in concentrations of all acids and ethanol (product of heterofermentative LAB metabolism) was observed in all samples; nevertheless significantly (*p* < 0.05) higher concentration of lactic, citric, and acetic acids was found at the beginning of storage in the sample with free cells. Tartaric acid was detected only in samples with raisins.

Both storage time and immobilization support (raisins), as well as their interaction, demonstrated a significant (*p* < 0.03) effect on acetic acid content in cheese. The highest concentrations of acetic acid were detected in the samples with raisins (not bearing *L. lactis* LL16 cells) at the beginning of cheese storage compared to the samples with immobilized cells on raisins. Our findings of further depletion of lactic and citric acids in all samples during storage is also supported by other authors, since it is now known that the end-product of lactose fermentation, lactate, can be catabolized further by some cheese LAB, mainly non-starter LAB [33] and the ability of LAB to use lactate and citrate for the production of flavor compounds has been confirmed [34]. Ethanol concentration in cheese was affected (*p* < 0.05) by immobilized cells (wet or dried), the storage time, and their interaction. Significantly (*p* < 0.05) lower ethanol yield was recorded in the sample with wet immobilized cells compared to the dry immobilized samples.

### 3.4. Volatiles in Cheese during Storage

The development of an intriguing aromatic profile is a desirable aim for the cheese industry [35]. Raisins, containing up to 5% of fructo-oligosaccharides, are known for a wide spectrum of various free form volatile compounds [36]. It is documented that wild type *L. lactis* is also contributing to volatiles in the fresh cheese matrix [37]. Hence, minor volatile by-products analysis was performed. All cheese samples were evaluated regarding their aromatic profile by headspace solid-phase microextraction gas chromatography-mass spectrometry (HS-SPME/GC-MS) analysis. A total of 54 volatile compounds were identified from cheese samples during storage, using HS-SPME extraction method and GC–MS analysis: 5 esters, 8 acids, 8 alcohols, 18 carbonyl compounds, and 15 miscellaneous compounds were found. The addition of raisins enhanced the volatile profile of cheeses with 2-furan-methanol, 1-octanol, 3-methylbutanal, 2-methylbutanal, 2-furancarboxaldehyde, 1-(2-furanyl)-ethanone, 5-methyl-2-furancarboxaldehyde. In general, all volatile compound groups, except organic acids, decreased during the storage of fresh cheeses. Alcohols accounted for the most (%) of the volatile profile, followed by carbonyl and miscellaneous compounds. Other authors [34] studying the impact of LAB on volatile profile of cheese also claim that among alcohols, ethanol was found at high relative percentages (10%) and that ethanol mainly originates from acetaldehyde in the lactate metabolic pathway.

**Figure 1:**
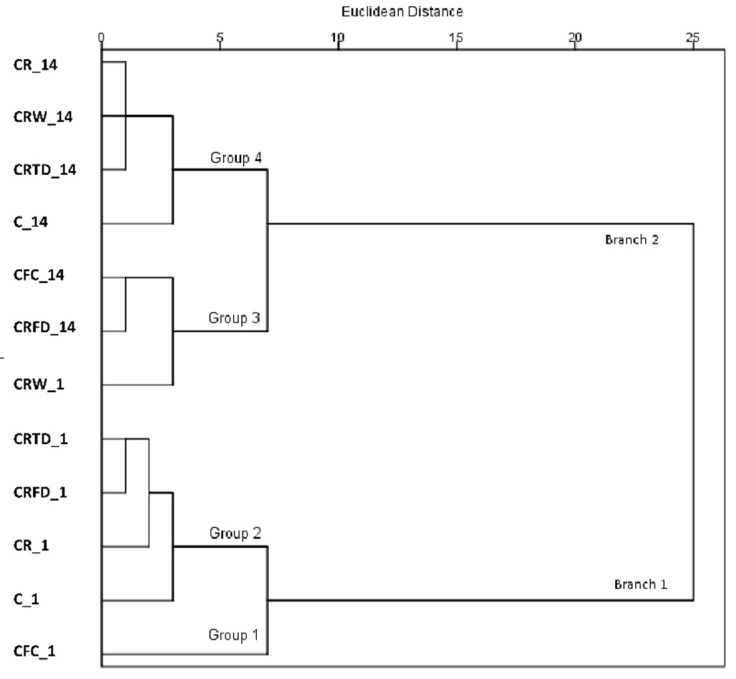
Dendrogram for the hierarchical cluster analysis (HCA) results of minor volatile compounds during storage days 1 and 14 of sweet curd cheese with *Lactococcus lactis* LL16 cells immobilized on raisins. Samples: control cheese (C); cheese with free cells (C + FC); cheese with raisins (C + R); cheese with wet immobilized cells on raisins (C + RW); cheese with freeze-dried immobilized cells on raisins (C + RFD); cheese with thermally dried immobilized cells on raisins (C + RTD).

A hierarchical cluster analysis (HCA) was applied to provide exploratory results on the volatile compound data. A two-dimensional diagram (dendrogram) was used to present agglomerated variables in a hierarchical form (Figure 1). For all cheese samples, HCA was used to distinguish concentrated groups of similar volatile compound profiles on the 1st and 14th days of storage. Figure 1 shows a structure of two branches subdivided into 4 groups and a series of smaller agglomerates. Branch 1 is defined by the cheeses on 1st day of storage. Branch 2 consists of all cheeses analyzed on the 14th day of storage and C + RW cheese on the 1st day of storage. Therefore, it is observed that, except for cheese made with wet cells immobilized on raisins, the storage period had the most effect on the volatile compound profile of cheeses. Exclusive volatile profile of C + RW sample at the 1st day of storage was most likely caused by the advanced activity of *L. lactis* cells during the storage time of wet immobilized cells on raisins at 4 °C for equal time period (24 h) that it took to prepare thermally and freeze dried immobilized cells (as discussed above). The results of moisture (Table 1) and sugars contents (Table 2) confirm that on the 1st day of storage C + RW sample had significantly higher moisture content and lower contents of lactose, glucose, fructose, and total sugars, compared to the rest of the samples, which indicates, that immobilized *L. lactis* LL16 cells were hydrolyzing sugars in raisins and afterwards contributing to a more enriched volatile profile of cheeses.

**Figure 2:**
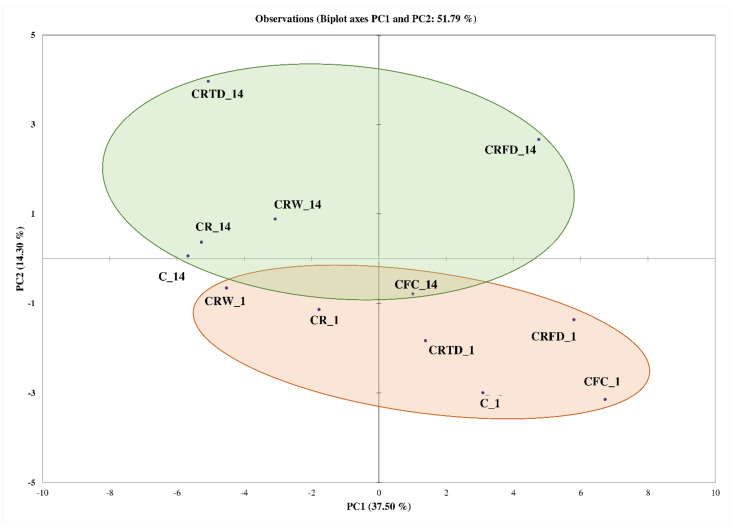
Score plot of the principal components (PC) of minor volatile compounds during storage days 1 and 14 of sweet curd cheese with *Lactococcus lactis* LL16 cells immobilized on raisins. Samples: control cheese (C); cheese with free cells (C + FC); cheese with raisins (C + R); cheese with wet immobilized cells on raisins (C + RW); cheese with freeze-dried immobilized cells on raisins (C + RFD); cheese with thermally dried immobilized cells on raisins (C + RTD).

The conducted PCA analysis of volatile profiles showed similar patterns. The PCA score plot (Figure 2) is in accordance with the results observed for HCA. The two principal components (PC1 and PC2) presented variance values of 37.5% and 14.3%, respectively. The full data of determined volatile profiles of cheese samples during storage is provided in the Supplementary material file (Figure S1).

### 3.5. Preliminary Sensory Evaluation

**Figure 3:**
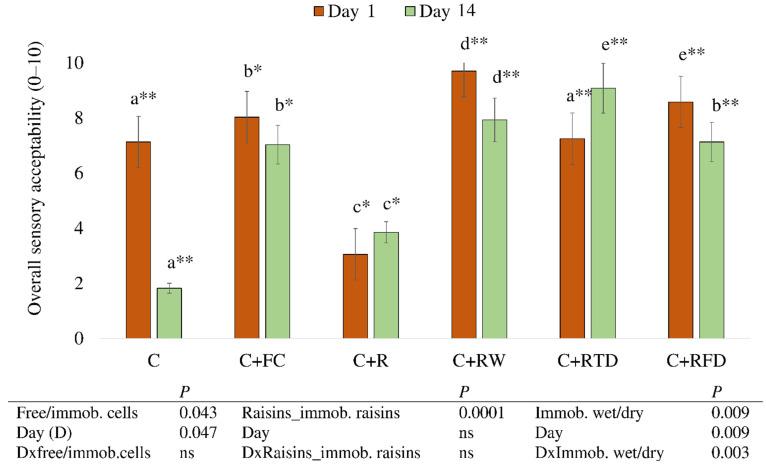
Overall sensory acceptability of sweet curd cheese with *Lactococcus lactis* LL16 cells immobilized on raisins on days 1 and 14 of storage. Samples: control cheese (C); cheese with free cells (C + FC); cheese with raisins (C + R); cheese with wet immobilized cells on raisins (C + RW); cheese with freeze-dried immobilized cells on raisins (C + RFD); cheese with thermally dried immobilized cells on raisins (C + RTD). No significant differences were found among samples with the same letters within the same day. Differences between storage days for the same sample were significant when *p* < 0.001 (*), *p* < 0.0001 (**). ns: not significant.

Raisins as a natural prebiotic were recently proposed as vehicles for immobilized probiotic strains delivering a fruity, distinctive flavor in dairy [21]. Overall sensory acceptability results of cheese samples are presented in Figure 3. Significant effects of single factors, such as the nature of cells (free versus immobilized cells), storage time (*p* < 0.05), immobilization support (raisins versus raisins with immobilized cells, *p* < 0.001), the drying method (wet versus dried), storage day (*p* < 0.01), as well as their interactions (*p* < 0.03) had a significant effect on overall acceptability of the experimental cheese. 

Due to rapidly developing off-flavors, the shelf life of traditional curd cheese is only 7–8 days. According to our previous studies [38,39], addition of *L. lactis LL16* to fresh cheese acts as a protective strain against spoilage bacteria, thus improving the flavor and prolonging the shelf-life of the product. In this study, we have also observed that *L. lactis* significantly increased the acceptability of cheese. Control cheeses were the least acceptable (C and C + R), while cheese samples with *L. lactis* (C + FC, C + RW, C + RFD), which had the highest lactococci counts on day 1 (Table 1), expressed the highest scores in overall sensory acceptability among all samples on the 1st storage day. The decrease in acceptability was observed in most of the samples at the end of the storage time. Only the addition of thermally dried raisins with immobilized *L. lactis* LL16 cells resulted in an increased cheese sensory acceptability during storage. This finding might me influenced by a significant increase in total sugars and volatile compounds in C + RTD sample.

## 4. Conclusions

The latest trend in the development of probiotic enriched food product is the usage of wild-type, indigenous novel potential probiotics isolated from various local sources. Moreover, the use of local and natural prebiotics as a support for the viability of such bacteria is highly encouraged by the consumers. Therefore, this study evaluated the possibility of enriching fresh sweet curd cheese with indigenous *Lactococcus lactis* strain immobilized on raisins and subsequently dried by various methods (wet, thermal drying, freeze-drying). 

A total of 54 volatile compounds were identified from cheese samples during storage, using HS-SPME extraction method and GC–MS analysis: 5 esters, 8 acids, 8 alcohols, 18 carbonyl compounds, and 15 miscellaneous compounds were found. The addition of raisins enhanced the volatile profile of cheeses with 2-furanmethanol, 1-octanol, 3-methylbutanal, 2-methylbutanal, 2-furancarboxaldehyde, 1-(2-furanyl)-ethanone, 5-methyl-2-furancarboxaldehyde. 

Microbiological analysis revealed that all immobilized cells demonstrated steady gradual growth in lactococci counts throughout the storage of cheese with thermally dried cells expressing the lowest counts, compared to wet and freeze-dried cells. Even though the growth of lactococci was slower in cheese samples with thermally dried immobilized cells on raisins, preliminary overall sensory evaluation results indicated that this supplementation was the most acceptable by the end of the cheese storage. Therefore, in future studies, lower thermal drying temperature and shorter drying time-period should be considered in order to increase the viability of probiotic cells.

The obtained results are encouraging for the production of functional cheeses with improved sensorial and nutritional characteristics on industrial and/or small industrial scale, since both investigated drying methods (freeze-drying and thermal drying) of raisins with immobilized *L. lactis* cells showed potential technological application. 

## Figures and Tables

**Table 1:** Physicochemical and microbiological parameters determined during the storage of sweet curd cheese with *Lactococcus lactis* LL16 immobilized on raisins.

Day	Sample ^1^	pH	a_w_	Moisture	TBC	Lactococci
1	C	5.93 ± 0.01 1Aa	0.93 ± 0.00 a	62.38 ± 0.13 Aa	5.79 ± 0.46 Aa	3.10 ± 0.71 a
	C + FC	5.94 ± 0.02 Aa	0.93 ± 0.01 a	64.28 ± 0.28 Ab	9.34 ± 0.04 Ab	8.79 ± 0.13 Ab
	C + R	5.54 ± 0.05 b	0.89 ± 0.00 b	57.13 ± 0.28 Ac	4.99 ± 0.12 ac	3.02 ± 0.03 ad
	C + RW	5.74 ± 0.01 c	0.90 ± 0.00 c	63.99 ± 0.13 Ad	6.39 ± 0.08 Ad	5.99 ± 0.13 Acd
	C + RTD	5.71 ± 0.04 c	0.91 ± 0.00 d	57.04 ± 0.24 Ac	5.42 ± 0.12 Aa	4.04 ± 0.062 Aad
	C + RFD	5.72 ± 0.01 Ac	0.91± 0.00 d	57.17 ± 0.21 Ac	5.69 ± 0.08 Aa	5.00 ± 0.01 Ad
7	C	6.01 ± 0.01 Ba	0.90 ± 0.04 a	62.40 ± 0.09 A	3.22 ± 0.59 Ba	2.69 ± 0.55 a
	C + FC	5.89 ± 0.03 Ab	0.89 ± 0.04 b	64.29 ± 0.07 A	9.40 ± 0.09 Ab	10.28 ± 0.01 Bb
	C + R	5.40 ± 0.06 c	0.86 ± 0.05 ce	55.93 ± 0.21 B	4.53 ± 0.55 a	3.33 ± 0.74 Ba
	C + RW	5.74 ± 0.01 d	0.89 ± 0.05 b	64.77 ± 0.13 B	6.18 ± 0.02 Ac	6.31 ± 0.01 Ac
	C + RTD	5.76 ± 0.01 d	0.88 ± 0.05 d	61.16 ± 0.15 B	5.12 ± 0.03 Aac	5.09 ± 0.09 ABc
	C + RFD	5.63 ± 0.01 Be	0.87 ± 0.06 ec	56.21 ± 0.16 B	6.40 ± 0.24 Ac	6.51 ± 0.27 Bc
14	C	5.89 ± 0.03 Aa	0.89 ± 0.05	64.76 ± 0.01 Ba	4.87 ± 0.02 ABa	3.63 ± 0.10 a
	C + FC	5.66 ± 0.03 Bab	0.87 ± 0.07	61.79 ± 0.01 Bb	10.43 ± 0.01 Bb	9.50 ± 0.03 Cb
	C + R	5.48 ± 0.16 b	0.89 ± 0.03	58.37 ± 0.01 Cc	6.03 ± 0.78 ac	3.78 ± 0.68 a
	C + RW	5.68 ± 0.03 ab	0.89 ± 0.04	63.27 ± 0.03 Cd	8.53 ± 0.53 Bd	7.82 ± 0.30 Bc
	C + RTD	5.60 ± 0.08 ab	0.88 ± 0.03	58.19 ± 0.01 Ce	7.41 ± 0.16 Bdc	6.36 ± 0.06 Bd
	C + RFD	5.54 ± 0.01 Ca	0.90 ± 0.01	59.12 ± 0.01 Cf	8.56 ± 0.47 Bd	7.90 ± 0.4 Cc
Significance of factors ^2^ and their interactions						
	FC/IC	0.000	ns	0.031	0.000	0.000
	Day	0.000	ns	ns	0.000	0.005
	Day × FC/IC	0.041	ns	ns	ns	0.052
	R/RIC	0.0001	ns	0.054	0.0001	0.0001
	Day	ns	ns	ns	0.0001	0.010
	Day × R/RIC	ns	ns	ns	ns	ns
	RW/RD	0.027	ns	0.0001	0.051	0.027
	Day	0.012	ns	ns	0.0001	0.001
	Day × RW/RD	ns	ns	ns	ns	ns

a_w_, water activity (%). Moisture, %. TBC, total bacterial count (log cfu/g). Lactococci (log cfu/g). ^1^ Samples: control cheese (C); cheese with free cells (C + FC); cheese with raisins (C + R); cheese with cells immobilized on wet raisins (C + RW); cheese with cells immobilized on freeze-dried raisins (C + RFD); cheese with cells immobilized on thermally dried raisins (C + RTD). SD: standard deviation, n = 3. ns: not significant. Means with different capital letters indicate significant differences (*p* < 0.05) among storage days for each cheese sample. Means with different lowercase letters indicate significant differences (*p* < 0.05) between cheese samples on the same storage day. ^2^ Factors: free cells (FC); immobilized cells (IC); raisins (R); raisins with immobilized cells (RIC); wet raisins with immobilized cells (RW); dried raisins with immobilized cells (RD).

**Table 2:** The profile of sugars and organic acids determined during the storage of sweet curd cheese with *L. lactis* LL16 cells immobilized on raisins.

		Sugars (g/100 g)					Organic Acids and Metabolites of Sugar Conversion (g/100 g)						
Day	Sample ^1^	Lactose	Galactose	Glucose	Fructose	Total sugars	Citric	Lactic	Acetic	Tartaric	Butyric	Glycerol	Ethanol
1	C	6.29 Aa	0.39 A	0.00	0.00	6.69 Aa	0.69 Aa	0.69 Aa	0.07 Aa	0.00 a	0.04 A	0.08 Aa	0.06 Aa
	C + FC	6.99 Ab	0.59 A	0.00	0.00	7.59 Ab	0.99 Ab	0.89 Ab	0.14 Ab	0.00 a	0.03 A	0.16 b	0.08 Aa
	C + R	6.89 Ac	0.49 A	11.30 A	12.90 A	31.19 Ac	0.79 Ac	0.99 Ac	0.13 Ab	1.10 Ab	0.00	0.60 Ac	0.07 Aa
	C + RW	3.99 Ad	0.00	6.19 A	6.89 A	17.09 Ad	0.59 Ad	0.39 Ad	0.02 Ad	0.60 Ac	0.00	0.16 Ab	0.03 b
	C + RTD	5.59 Ae	0.00	11.40 A	13.30 A	30.49 Ae	0.79 Ac	0.79 Ae	0.06 Aa	1.20 b	0.00	0.28 Ad	0.09 Ab
	C + RFD	4.39 Af	0.00	11.70 A	13.50 A	29.79 Af	0.59 Ad	0.59 Af	0.05 Aa	1.60 Ad	0.00 A	0.23 Ae	0.07 Ac
	SD	1.27	0.28	5.63	6.50	11.57	0.15	0.22	0.05	0.66	0.02	0.18	0.02
7	C	5.69 Ba	0.49 B	0.00	0.00	6.09 Ba	0.79 Ba	0.79 Ba	0.14 a	0.00	0.02	0.13 Ba	0.05 Aa
	C + FC	6.09 Bb	0.49 B	0.00	0.00	6.59 Bb	0.89 Bb	0.79 Ba	0.12 a	0.00	0.02	0.11 Ba	0.07 ABa
	C + R	4.69 Bc	0.29 B	10.10 B	11.50 B	26.49 Bc	0.59 Bc	0.69 Bb	0.08 Bb	1.20 AB	0.00	0.36 Bb	0.02 Bb
	C + RW	4.19 Bd	0.00	6.39 B	7.29 B	17.99 Bd	0.49 Bd	0.39 Bc	0.05 Bb	0.90 B	0.00	0.17 Bc	0.02 b
	C + RTD	4.69 Bc	0.00	4.09 B	4.79 BB	13.59 Be	0.49 Bd	0.39 Bc	0.03 Bd	0.50	0.00	0.13 Ba	0.04 Ba
	C + RFD	5.49 Be	0.00	10.40 B	12.20 B	28.29 Bf	0.69 Be	0.69 Bd	0.06 b	1.20 B	0.35 B	0.26 Bd	0.04 Ba
	SD	0.73	0.25	4.64	5.36	9.55	0.16	0.19	0.04	0.55	0.14	0.10	0.02
14	C	5.39 Ca	0.29 C	0.00	0.00	5.69 Ca	0.59 Ca	0.49 Ca	0.02 Ca	0.00	0.00 B	0.04 Ca	0.01 Ba
	C + FC	5.49 Cb	0.39 C	0.00	0.00	5.79 Cb	0.59 Ca	0.69 Cb	0.07 Bb	0.00	0.00 B	0.05 Ca	0.03 Ba
	C + R	4.79 Cc	0.00 C	7.59 C	8.59 C	20.99 Cc	0.49 Cb	0.49 Ca	0.03 Ca	0.60 B	0.00	0.26 Cb	0.04 ABb
	C + RW	4.59 Cd	0.00	4.09 C	4.89 C	13.59 Cd	0.59 Aa	0.29 Ac	0.03 ABa	0.50 A	0.00	0.12 Ac	0.01 a
	C + RTD	5.49 Cb	0.00	4.99 CC	5.89	16.39 Ce	0.49 Bb	0.39 Bd	0.03 Ba	0.50	0.00	0.13 Bc	0.00 Ca
	C + RFD	4.49 Ce	0.00	6.89 C	7.99 C	19.29 Cf	0.49 Cb	0.39 Cd	0.04 Ca	0.40 C	0.00 A	0.14 Cc	0.00 Ca
	SD	0.47	0.18	3.29	3.78	6.61	0.05	0.14	0.02	0.27	0.01	0.08	0.02
Significance of factors ^2^													
	FC/IC	0.021		0.006	0.007	0.016	0.010	0.024	0.001	0.013	ns	ns	ns
	Day	ns		ns	ns	ns	0.045	ns	0.041	ns	ns	ns	0.033
	Day × FC/IC	ns		ns	ns	ns	ns	ns	ns	ns	ns	ns	ns
	R/RIC	ns		ns	ns	ns	ns	ns	0.009	ns	ns	0.001	ns
	Day	ns		ns	ns	ns	ns	ns	0.013	ns	ns	0.006	ns
	Day × R/RIC	ns		ns	ns	ns	ns	ns	0.031	ns	ns	ns	0.05
	RW/RD	ns		ns	ns	ns	ns	ns	ns	ns	ns	ns	0.041
	Day	ns		ns	ns	ns	ns	ns	ns	ns	ns	ns	0.013
	Day × RW/RD	ns		ns	ns	ns	ns	ns	ns	ns	ns	ns	0.054

^1^ Samples: control cheese (C); cheese with free cells (C + FC); cheese with raisins (C + R); cheese with cells immobilized on wet raisins (C + RW); cheese with cells immobilized on freeze-dried raisins (C + RFD); cheese with cells immobilized on thermally dried raisins (C + RTD). SD: standard deviation, n = 3. ns: not significant. SD: standard deviation, n = 3. ns: not significant. Means with different capital letters indicate significant differences (*p* < 0.05) among storage days for each cheese sample. Means with different lowercase letters indicate significant differences (*p* < 0.05) between cheese samples on the same storage day. ^2^ Factors: free cells (FC); immobilized cells (IC); raisins (R); raisins with immobilized cells (RIC); wet raisins with immobilized cells (RW); dried raisins with immobilized cells (RD).

## Data Availability

Data is contained within the article and supplementary materials.

## Supplementary Materials

The following are available online at https://www.mdpi.com/article/10.3390/foods11091311/s1, Figure S1: Graphical illustration of production of fresh curd cheese; Figure S2: Graphical illustration of preparation of experimental cheese samples; Table S1: Minor volatile compounds (%) identified in curd cheese fortified with free or immobilized (wet or dried) *Lactococcus lactis* cells on raisins. References [25,40,41,42,43,44,45,46] cited in Supplementary Materials.
