# Supplementary material for: Indigenous Lactococcus lactis with Probiotic Properties: Evaluation of Wet, Thermally- and Freeze-Dried Raisins as Supports for Cell Immobilization, Viability and Aromatic Profile in Fresh Curd Cheese
Source: Foods. 2022 Apr 30;11(9):1311. doi: 10.3390/foods11091311 (PMC9101569; doi:10.3390/foods11091311)
Supplement: Supplementary file 1 [file foods-11-01311-s001.zip › foods-1686680-supplementary.pdf]

Supplementary Materials

Figure S1. Graphical illustration of production of fresh curd cheese

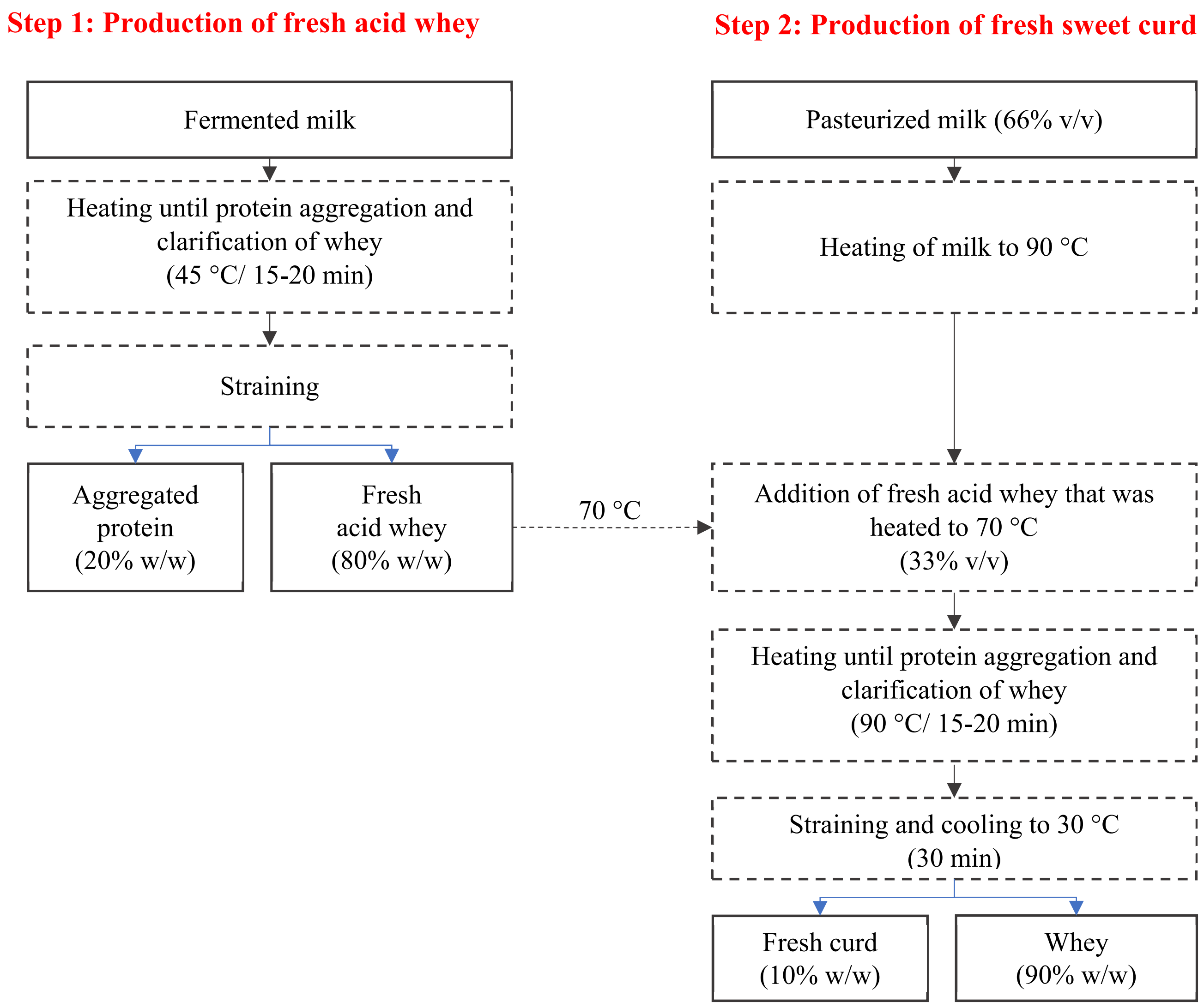

Figure S2. Graphical illustration of preparation of experimental cheese samples: control cheese (C); cheese with free cells (C+FC); cheese with raisins (C+R); cheese with cells immobilized on wet raisins (C+RW); cheese with cells immobilized on freeze-dried raisins (C+RFD); cheese with cells immobilized on thermally dried raisins (C+RTD).

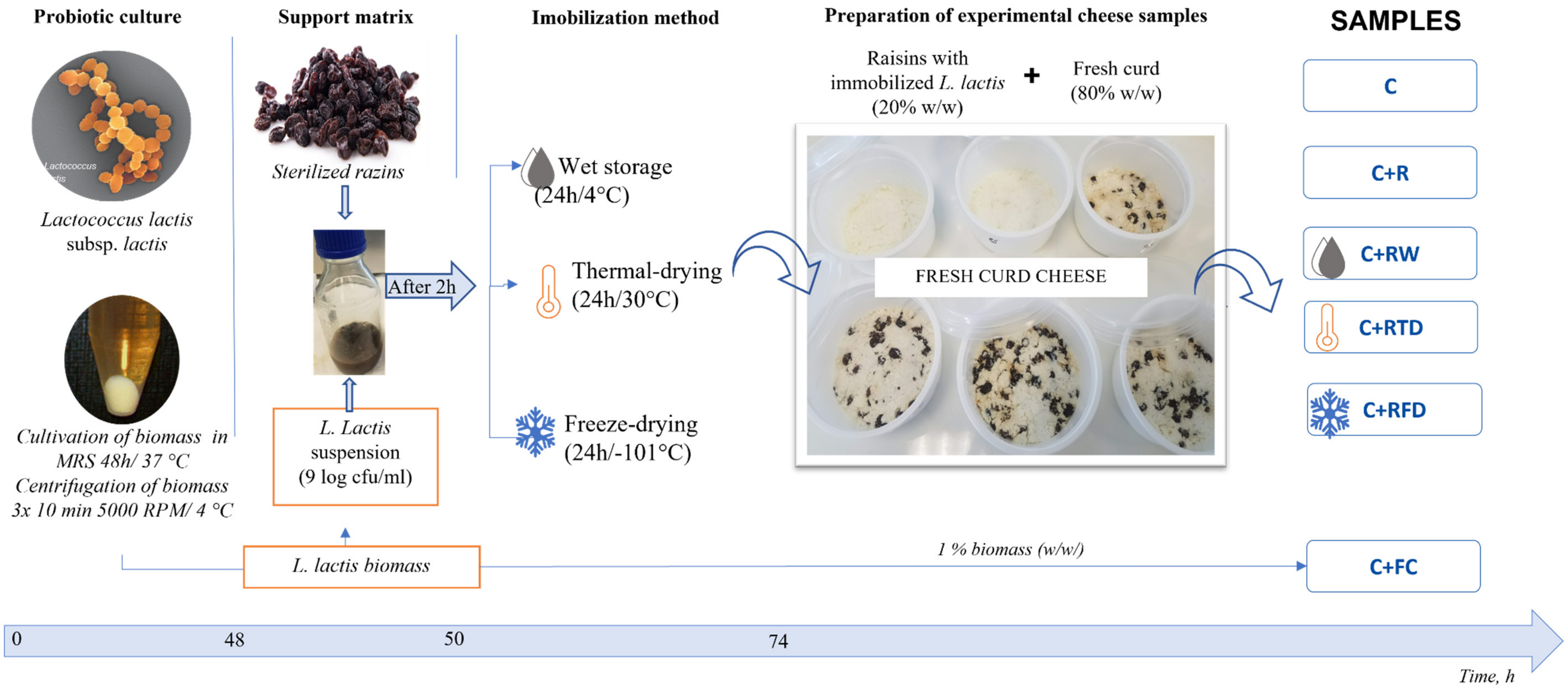

Table S1. Minor volatile compounds (%) identified in curd chesses fortified with free or immobilized (wet or dried) *Lactococcus lactis* cells on raisins

Storage day			Day 1						Day 14					
Esters	Kovats' index	Kovats' index ref	C	C+FC	C+R	C+RW	C+RTD	C+RFD	C	C+FC	C+R	C+RW	C+RTD	C+RFD
Ethyl acetate	609	612[a]	2.4	10.5	6.1	3.7	7.6	28.5	5.7	7.5	4.1	4.7	12.4	5.2
Ethyl butanoate	824	803[b]	4.0	10.6	2.5	2.1	ND	ND	1.3	8.6	1.9	5.1	2.3	4.1
Butyl acetate	834	812[b]	2.9	ND	1.3	1.4	ND	8.1	ND	3.6	0.9	1.9	ND	3.2
2-methyl-1-butanol acetate	887	869[b]	ND	ND	ND	ND	ND	2.9	3.5	5.6	1.6	2.4	1.4	8.3
ethyl hexanoate	1015	1002[b]	4.5	9.5	3.6	3.5	3.4	5.8	1.2	4.3	1.5	3.6	ND	ND
Total esters			**13.8**	**30.6**	**13.5**	**10.8**	**11.0**	**45.2**	**11.8**	**29.7**	**10.0**	**17.7**	**16.1**	**20.8**
Organic acids														
Acetic acid	608	602[d]	ND	ND	ND	ND	ND	ND	ND	ND	1.7	ND	ND	ND
Butanoic acid	776	793[d]	7.5	34.8	3.4	3.1	9.1	18.1	2.4	17.7	2.3	5.1	5.4	13.7
Hexanoic acid	1012	1003[c]	53.6	152.5	26.8	20.0	36.4	85.3	16.3	69.3	16.5	24.6	32.4	109.3
Octanoic acid	1193	1198[b]	83.1	163.1	20.7	19.2	24.2	85.5	14.6	50.5	13.5	30.6	26.9	109.9
Nonanoic acid	1286	1291[c]	6.3	10.3	6.9	4.5	2.6	20.3	0.9	ND	3.8	4.5	9.5	51.9
Decanoic acid	1378	1381[b]	23.8	52.1	3.9	3.6	4.5	9.9	2.7	ND	23.7	3.3	11.4	62.7
Hexadecanoic acid	1967	1964[e]	ND	ND	2.1	1.1	ND	ND	0.5	ND	0.8	ND	8.0	1.6
Octadecanoic acid	>2100	2172[e]	ND	ND	1.6	1.3	ND	ND	1.1	ND	2.1	ND	12.6	2.4
Total organic acids			**174.3**	**412.7**	**65.4**	**52.8**	**76.8**	**219.1**	**38.4**	**137.5**	**64.4**	**68.1**	**106.1**	**351.5**
Alcohols														
Ethanol	<600	537[e]	6239.3	7861.9	5327.6	4027.2	5636.4	5007.6	1825.0	3501.5	3072.1	3000.9	2678.2	3939.7
1-methoxy-2-propanol	629		ND	ND	ND	ND	ND	ND	1.0	6.2	5.6	20.5	3.1	ND
3-methyl-1-butanol	712	734[e]	3.1	10.2	1.7	ND	3.2	6.5	1.3	10.3	ND	2.2	ND	ND
2,3-butanediol	731	793[g]	ND	ND	ND	ND	ND	ND	ND	ND	ND	ND	ND	ND
2-Furanmethanol	870	864[f]	ND	ND	ND	ND	ND	ND	ND	ND	1.2	6.7	0.7	3.2
1-hexanol	881	861[b]	ND	ND	ND	1.4	ND	5.6	ND	5.1	ND	6.7	ND	2.2
2,2'oxybis-ethanol	985		ND	ND	ND	ND	ND	ND	ND	ND	ND	ND	79.5	ND
1-octanol	1093	1076[c]	ND	ND	0.8	0.8	2.7	1.8	ND	ND	ND	0.9	1.0	3.4
Benzothiazole	1220	1227[g]	17.9	47.4	17.7	7.8	58.9	46.6	10.6	30.9	9.0	11.6	7.2	17.6
Total alcohols			**6260.3**	**7919.4**	**5347.9**	**4037.1**	**5701.2**	**5068.2**	**1837.9**	**3554.1**	**3087.9**	**3049.4**	**2769.7**	**3966.1**
Carbonyl compounds														
3-methyl butanal	641	651[f]	0.0	0.0	32.4	3.7	24.9	71.0	0.0	0.0	8.8	2.6	3.8	17.5
2-methyl butanal	654	662[f]	0.0	0.0	12.2	1.8	7.9	23.8	0.0	0.0	3.6	0.0	0.0	5.5
2-pentanone	686	686[f]	2.3	11.3	1.6	0.6	3.3	7.8	1.0	0.0	0.0	3.0	0.0	0.0
3-hydroxy-2-butanone	723	720[e]	16.3	59.1	14.9	8.7	18.5	55.9	6.6	37.0	5.6	13.5	4.7	9.5
Hexanal	823	800[e]	16.3	17.2	11.0	5.7	28.0	33.4	4.5	10.8	5.6	6.6	8.1	10.2
2-Furancarboxaldehyde	846	838[g]	0.0	0.0	402.8	80.4	53.6	160.6	0.0	0.0	202.9	20.0	25.7	147.9
2-Heptanone	896	890[c]	38.8	72.2	18.0	12.8	36.6	44.9	9.0	34.0	6.4	18.5	7.3	30.4
Heptanal	906	901[e]	2.8	3.1	1.8	1.2	2.7	3.1	0.7	1.3	0.8	1.3	1.2	3.9
1-(2-furanyl)-ethanone	915	914[h]	0.0	0.0	11.2	1.3	0.0	4.1	0.0	0.0	6.6	1.7	1.3	6.3
Benzaldehyde	964	947[b]	2.0	1.4	2.8	2.7	3.6	4.6	0.5	0.0	1.5	1.2	1.6	6.7
5-methyl-2-furancarboxaldehyde	971	964[e]	0.0	0.0	14.7	3.3	2.8	4.5	0.0	0.0	8.6	1.5	2.3	9.6
2-nonanone	1126	1096[c]	12.0	16.2	4.9	3.0	8.9	9.9	1.9	6.0	1.4	4.2	3.5	11.8
Nonanal	1135	1108[c]	17.4	18.5	11.0	8.3	34.9	21.7	5.0	10.2	6.6	11.0	14.6	25.4
2,3-dihydroxy-3,5-dihydroxy-6-methyl-4H-Pyran-4-one	1163	1149[g]	0.0	0.0	0.0	0.0	0.0	0.0	0.0	0.0	0.0	0.0	3.9	0.0
Decanal	1206	1208[c]	4.9	7.9	2.5	2.7	3.5	9.3	1.1	4.1	1.4	2.5	3.1	10.4
5-(hydroxymethyl)-2-furancarboxaldehyde	1244	1252[g]	0.0	0.0	0.0	0.0	0.0	0.0	0.0	0.0	0.0	0.0	8.0	16.1
2-undecanone	1303	1310[c]	6.1	5.0	2.9	2.3	4.7	6.2	0.9	3.2	1.1	2.3	2.5	8.0
5,6,7,7a-tetrahydro-4,4,7a-trimethyl-2(4H)-benzofuranone	1417	1525[e]	12.1	8.4	4.8	2.8	5.8	9.9	4.3	10.9	2.8	5.6	3.8	15.0

Total carbonyl compounds			**130.9**	**220.4**	**549.6**	**141.1**	**239.9**	**470.9**	**35.7**	**117.5**	**263.6**	**95.4**	**95.5**	**334.0**
Miscellaneous compounds														
Hexane	600		751.5	397.2	837.6	5.0	15.9	31.4	146.7	383.7	56.3	146.9	153.8	495.8
Toluene	805	773[e]	27.4	20.5	14.0	17.5	16.7	17.5	10.9	25.5	12.3	11.0	2.7	15.0
2,4-dimethyl-heptane	836		ND	ND	ND	6.5	ND	ND	ND	ND	ND	ND	ND	ND
Ethylbenzene	866	857[a]	3.0	5.1	1.5	0.8	2.6	3.6	0.5	1.9	1.2	0.7	0.4	2.1
p-xylene	872	865[a]	20.9	28.1	9.4	4.7	13.3	18.7	3.4	13.6	4.9	7.3	2.8	16.2
1,2-dimethylbenzene	893	894[e]	3.4	4.6	1.5	0.8	2.3	2.6	0.6	1.7	0.6	1.3	0.6	1.9
1,3,5-trimethyl-benzene	972	956[b]	3.6	1.4	ND	ND	ND	ND	1.0	2.3	ND	ND	ND	ND
D-Limonene	1040	1025[a]	6.3	18.4	6.8	4.5	5.5	16.9	3.5	10.4	7.6	5.6	16.1	52.1
3,5,24-trimethyl tetracontane	1118	1172	15.0	12.5	9.4	3.0	15.7	14.4	3.9	11.5	4.0	7.0	6.5	11.4
N-ethyl benzenamine	1152		109.8	58.4	65.7	26.4	105.6	99.0	28.4	79.4	34.2	54.2	28.7	73.5
Dodecane	1200		8.3	11.7	3.8	2.6	4.4	8.0	1.6	6.2	2.0	3.8	4.1	10.8
1,3-bis(1,1-dimethylethyl)-benzene	1260	1267[c]	ND	ND	3.4	ND	ND	6.9	ND	3.0	ND	ND	ND	ND
Tetradecane	1400	1399[c]	7.3	7.3	3.9	2.4	5.1	10.1	1.8	6.7	2.2	3.5	4.2	9.1
Pentadecane	1505		5.4	8.1	8.8	1.8	4.2	8.0	2.0	7.3	2.2	2.6	1.9	6.2
Butylated Hydroxytoluene	1519	1522[c]	839.8	682.9	404.0	259.6	475.4	674.1	303.5	1150.0	269.9	577.3	184.3	1005.4
Total miscellaneous compounds			**1801.6**	**1256.3**	**1369.7**	**335.6**	**669.7**	**911.1**	**507.8**	**1703.3**	**397.3**	**822.5**	**408.3**	**1699.4**
Total volatile compounds			**8381.0**	**9839.5**	**7346.0**	**4577.5**	**6695.8**	**6714.5**	**2431.5**	**5542.0**	**3823.3**	**4051.6**	**3393.4**	**6371.8**

Samples: control cheese (C); cheese with free cells (C+FC); cheese with raisins (C+R); cheese with cells immobilised on wet raisins (C+RW); cheese with cells immobilised on freeze-dried raisins (C+RFD); cheese with cells immobilised on thermally dried raisins (C+RTD). ND: not detected.
